# The human ABCB6 protein is the functional homologue of HMT-1 proteins mediating cadmium detoxification

**DOI:** 10.1007/s00018-019-03105-5

**Published:** 2019-05-03

**Authors:** Zsófia Rakvács, Nóra Kucsma, Melinda Gera, Barbara Igriczi, Katalin Kiss, János Barna, Dániel Kovács, Tibor Vellai, László Bencs, Johannes M. Reisecker, Norbert Szoboszlai, Gergely Szakács

**Affiliations:** 1 Institute of Enzymology, Research Centre for Natural Sciences, Hungarian Academy of Sciences, Budapest, Hungary; 2 Department of Genetics, Institute of Biology, Eötvös Loránd University, Budapest, Hungary; 3 Department of Analytical Chemistry, Institute of Chemistry, Eötvös Loránd University, Budapest, Hungary; 4 Institute for Solid State Physics and Optics, Wigner Research Centre for Physics, Hungarian Academy of Sciences, Budapest, Hungary; 5 Department of Medicine I, Comprehensive Cancer Center, Institute of Cancer Research, Medical University of Vienna, Vienna, Austria

**Keywords:** ABC transporters, Subcellular localization, ABCB6, HMT-1, Endolysosomal system, Detoxification, Cadmium

## Abstract

ABCB6 belongs to the family of ATP-binding cassette (ABC) transporters, which transport various molecules across extra- and intra-cellular membranes, bearing significant impact on human disease and pharmacology. Although mutations in the *ABCB6* gene have been linked to a variety of pathophysiological conditions ranging from transfusion incompatibility to pigmentation defects, its precise cellular localization and function is not understood. In particular, the intracellular localization of ABCB6 has been a matter of debate, with conflicting reports suggesting mitochondrial or endolysosomal expression. ABCB6 shows significant sequence identity to HMT-1 (heavy metal tolerance factor 1) proteins, whose evolutionarily conserved role is to confer tolerance to heavy metals through the intracellular sequestration of metal complexes. Here, we show that the cadmium-sensitive phenotype of *Schizosaccharomyces pombe* and *Caenorhabditis elegans* strains defective for HMT-1 is rescued by the human ABCB6 protein. Overexpression of ABCB6 conferred tolerance to cadmium and As(III) (As_2_O_3_), but not to As(V) (Na_2_HAsO_4_), Sb(V), Hg(II), or Zn(II). Inactivating mutations of ABCB6 abolished vacuolar sequestration of cadmium, effectively suppressing the cadmium tolerance phenotype. Modulation of ABCB6 expression levels in human glioblastoma cells resulted in a concomitant change in cadmium sensitivity. Our findings reveal ABCB6 as a functional homologue of the HMT-1 proteins, linking endolysosomal ABCB6 to the highly conserved mechanism of intracellular cadmium detoxification.

**Electronic supplementary material:**

The online version of this article (10.1007/s00018-019-03105-5) contains supplementary material, which is available to authorized users.

## Introduction

ATP-binding cassette (ABC) transporters constitute one of the largest protein families in prokaryotes and eukaryotes. ABC transporters are integral transmembrane proteins that function as active transporters, channels or regulators. Active ABC transporters harness the energy of ATP to move a diverse array of substrates in or out of cells, or into cellular vesicles. There are 48 human ABC transporters, many of which are linked to severe inherited diseases, such as cystic fibrosis, or X-linked adrenoleukodystrophy [1]. Whereas several human ABC transporters have dedicated physiological roles (e.g., transport of phosphatidylcholine by ABCB4/MDR3; antigen processing by ABCB2/3), most recognize various xenobiotics and contribute to the “chemoimmunity” network of cells and organisms [2]. At the cellular level, multidrug resistance (MDR) transporters such as P-glycoprotein (ABCB1) play an important role in cancer drug resistance by reducing the concentration of chemotherapeutics below a cell-killing threshold. In addition, MDR transporters are also expressed in pharmacological barriers such as the blood–brain barrier, where they modulate the passage of drugs [3].

ABCB6 is widely expressed in many tissues, especially in the heart, liver, skeletal muscles [4], the red blood cells [5, 6], and skin [7]. ABCB6 is a half transporter of 842 amino acids, containing a unique N-terminal region followed by the ABC core consisting of a transmembrane domain and a cytoplasmic nucleotide-binding domain. ABCB6 forms homodimers [8, 9] and was shown to possess ATPase and transport activities after purification and functional reconstitution into liposomes [10]. At present, the subcellular localization of ABCB6 remains a matter of debate. In 2006, ABCB6 was described as a mitochondrial porphyrin transporter with an essential role in heme biosynthesis [8]. Subsequent studies have found ABCB6 to be dispensable for erythropoiesis [5, 9], suggesting that mitochondrial porphyrin import may not depend on ABCB6. In addition, several research groups have identified ABCB6 in extramitochondrial compartments, challenging the paradigm linking the expression and function of ABCB6 to mitochondria. ABCB6 was detected in the plasma membrane of cells [11], the red blood cell membrane [5, 9], melanosomes [12] and throughout the endolysosomal continuum [13–17]. However, the physiological function of ABCB6 in the endolysosomal compartment has remained elusive.

ABCB6 exhibits topological and sequential similarity to HMT (Heavy Metal Tolerance) family proteins (Supplementary Table 1). HMT-1 proteins in fission yeast (*Schizosaccharomyces pombe*), nematode (*Caenorhabditis elegans*) and the fruit fly (*Drosophila melanogaster*) fulfill a conserved role in conferring heavy metal resistance [18–21]. In fission yeast, SpHMT-1 mediates the vacuolar sequestration of metal adducts including phytochelatin, glutathione or metallothionein complexes of heavy metal ions [18]. An elegant study from the Vatamaniuk laboratory has shown that HMT-1 proteins in *C. elegans* (CeHMT-1) and *D. melanogaster* (DmHMT-1) can also mediate the sequestration and elimination of Cd complexes. In particular, heterologously expressed DmHMT-1 or CeHMT-1 were shown to suppress the cadmium hypersensitivity of *S. pombe hmt*-*1* mutants, concomitant with the localization of CeHMT-1 to the vacuolar membrane. These results clearly indicated that the HMT-1-mediated detoxification of heavy metals is preserved during evolution, extending to some invertebrate species lacking the ability to synthesize phytochelatin (PC) [20, 21]. Given the similarity of HMT-1 and ABCB6 sequences, the major aim of this study was to test if ABCB6 can complement the function of HMT-1 proteins. We show that ABCB6 can be functionally expressed in the vacuolar/endosomal membrane, resulting in a rescue of the cadmium sensitivity phenotype of HMT-1-deficient *S. pombe* and *C. elegans* strains. Our findings reveal ABCB6 as a functional orthologue of the HMT-1 proteins, linking ABCB6 to the highly conserved mechanism of intracellular cadmium detoxification. Consistent with our previous findings showing extramitochondrial localization, these results provide functional evidence supporting the endolysosomal function of ABCB6.

## Results

### Heterologous expression of human ABCB6 restores cadmium tolerance of *S. pombe* hmt-1Δ mutants

**Fig. 1:**
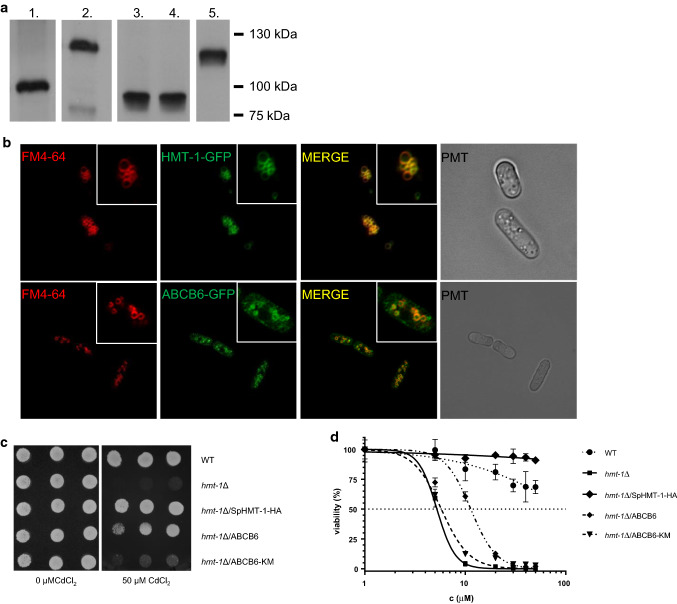
ABCB6 confers cadmium tolerance in *hmt*-*1*Δ mutant *S. pombe*. **a** SDS-PAGE and immunoblot analysis of yeast cells expressing HMT-1-HA (lane 1), HMT-1-GFP (lane 2), ABCB6 (lane 3), ABCB6-KM (lane 4) or ABCB6-GFP (lane 5). Expression of SpHMT-1 was revealed using anti-HA antibody; ABCB6 was labeled by the ABCB6-567 antibody; EGFP tagged proteins were labeled by an anti-EGFP antibody. **b** ABCB6-GFP (green) localizes to vacuoles (red) of *S. pombe*. *Hmt*-*1*-deleted *S. pombe* was transformed with pREP1-HMT-1-GFP or ABCB6-GFP; vacuoles were stained with FM 4–64. Insets show individual cells. Scale bar 10 μm. **c** Wild-type *S. pombe* cells transformed with empty pREP1 vector (WT); *hmt*-*1*Δ mutant cells transformed with empty pREP1 vector (*hmt*-*1*Δ), pREP1-HMT-1-HA (*hmt*-*1*Δ/SpHMT-1-HA), pREP1-ABCB6 (*hmt*-*1*Δ/ABCB6) or pREP1-ABCB6-KM (*hmt*-*1*Δ/ABCB6-KM) were grown overnight to an *A*_600nm_ of 1.8. Aliquots of the cell suspensions were then serially diluted and spotted onto solid EMM supplemented with adenine, uracil and the indicated concentrations of CdCl_2_. Colonies were visualized after incubating the plates for 8 days at 30 °C. **d** Transformants were grown overnight to an *A*_600nm_ of 0.8–1. Aliquots of 100-μL were inoculated into 2 mL of the same medium containing CdCl_2_ at the indicated concentrations. *A*_600nm_ was measured after growth at 30 °C for 72 h. Values, expressed as viability (%), were normalized to untreated control (*n* = 3)

To test whether ABCB6 and SpHMT-1 have overlapping functions, we expressed the wild-type human ABCB6 protein, a catalytically inactive mutant variant (ABCB6-KM [9]) and SpHMT-1 in a *hmt*-*1*-deleted mutant *S. pombe* strain showing increased cadmium (Cd) sensitivity (Fig. 1a). SpHMT-1-GFP was also localized to the vacuoles, matching the staining of the vacuolar membrane by FM 4–64 [22]. Confocal microscopy analysis of cells expressing ABCB6-GFP or SpHMT-1-GFP revealed a similar intracellular pattern, indicating that the human ABCB6 protein is targeted to the yeast vacuoles (Fig. 1b). As expected, expression of SpHMT-1 fully eliminated the increased cadmium sensitivity of the *hmt*-*1*Δ mutant strain. Expression of wild-type ABCB6 also restored cadmium tolerance, allowing transformed *S. pombe* colonies to grow in the presence of Cd(II) (Fig. 1c, Supplementary Figure 1). Rescue of *hmt*-*1*-deleted strains depended on the functionality of the heterologously expressed transporter, since an inactivating mutation affecting a conserved Walker A lysine of ABCB6 prevented the growth of *hmt*-*1*-deleted colonies in the presence of cadmium. Rescue was also observed in liquid medium (Fig. 1d). Cytotoxicity assays revealed that the expression of ABCB6 in *hmt*-*1*Δ *S. pombe* cells conferred resistance to As(III), but not to As(V), Sb(V), Hg(II), or Zn(II) (Supplementary Figure 2).

### Determination of vacuolar cadmium content

**Fig. 2:**
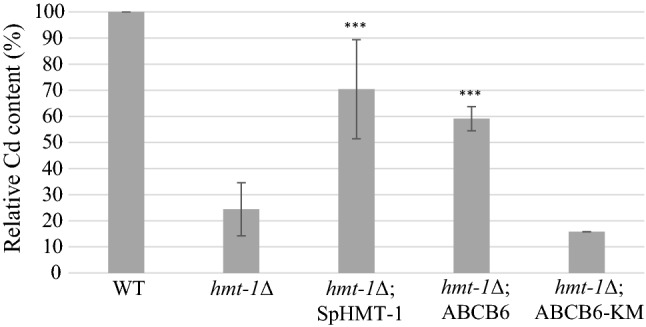
ABCB6 restores vacuolar Cd levels in *hmt*-*1*Δ mutant *S. pombe*. Yeast cells lacking SpHMT-1 (*hmt*-*1*Δ) were engineered to express ABCB6, ABCB6-KM or SpHMT-1. Transformed cells were cultured for 18 h in growth medium supplemented with 20 μM CdCl_2_. Intact vacuoles were isolated by differential centrifugation; the Cd content of the isolated vacuoles was quantified by GFAAS. Data show average Cd levels relative to control calculated from independent experiments (*n* = 3)

SpHMT-1 reduces the intracellular concentrations of cadmium by catalyzing the vacuolar sequestration of Cd–PC complexes [18]. To verify that the ability of ABCB6 to suppress the Cd hypersensitivity of HMT-1-deficient *S. pombe* mutants relies on an orthologous function, we assayed the Cd contents of intact vacuoles isolated from CdCl_2_-treated yeast cells. The integrity of the purified vacuoles was confirmed by acridine-orange (AO) staining (Supplementary Figure 3). Graphite furnace atomic absorption spectrometry (GFAAS) analysis showed that, as compared to the wild type, vacuoles isolated from *hmt*-*1*-deleted strains contained significantly lower amounts of cadmium, in line with the absence of vacuolar sequestration. Vacuolar cadmium levels were almost fully restored by the expression SpHMT-1 or ABCB6 (Fig. 2). Increased vacuolar accumulation of cadmium was dependent on the functionality of ABCB6, indicating that the rescue of *hmt*-*1*-deleted strains was due the ABCB6-mediated vacuolar sequestration of cadmium.

### Human ABCB6 rescues the Cd hypersensitivity of *hmt-1*-deleted *C. elegans*

**Fig. 3:**
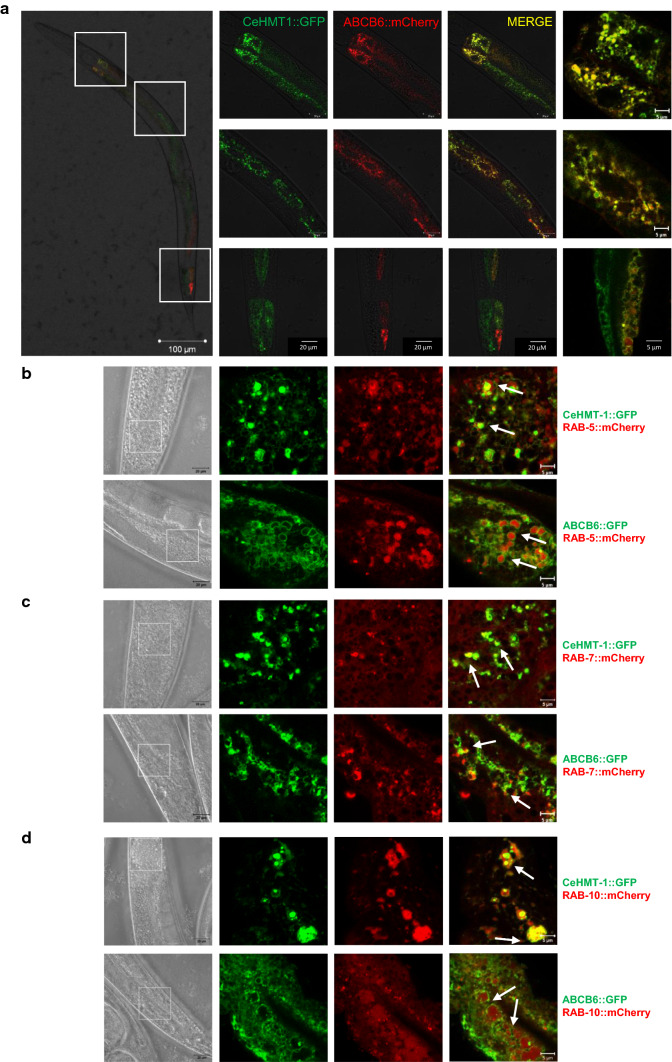
CeHMT-1 and ABCB6 show identical localization in nematodes. **a** Confocal microscopy images of an adult nematode co-expressing ABCB6::mCherry and CeHMT-1::GFP. Crossing of strains expressing CeHMT-1::GFP and ABCB6::mCherry proved that the two transporters are expressed in identical subcellular compartments (scale bar: 100 μm). Boxed areas are shown at a higher magnification (scale bar 20 and 5 μm). Note that the ABCB6::mCherry strain carries the transgene extrachromosomally, resulting in a mosaic expression of ABCB6. **b**–**d** CeHMT-1 and ABCB6 partially colocalize with markers of the early, late and basolateral recycling endosomes. Subcellular localization of ABCB6 and CeHMT-1 was determined by confocal microscopy. Strains expressing CeHMT-1::GFP or ABCB6::GFP were crossed with worms expressing RAB-5::mCherry (early endosomal marker, **b**) or RAB-7::mCherry (late and early endosomal marker, **c**), or RAB-10::mCherry (basolateral recycling endosomes marker, **d**). The panels show the DIC images (left) the GFP (green) and the mCherry (red) signals and the overlay of the two (right). Scale bar: 20 and 5 μm

CeHMT-1 is expressed in liver-like cells, the coelomocytes, as well as in head and tail neurons, and in the intestinal cells of *C. elegans* [21]. Crossing of strains expressing CeHMT-1::GFP and ABCB6::mCherry allowed the simultaneous evaluation of the subcellular expression of both proteins. Images obtained with confocal microscopy have indicated that ABCB6 is expressed in the same tissues as CeHMT-1 (Fig. 3a). To establish the subcellular localization of CeHMT-1 and ABCB6, we performed colocalization experiments using a lysosomal marker [24]. Interestingly, the intracellular organelles corresponding to the sites of CeHMT-1 or ABCB6 expression proved to be distinct from Lysotracker Red-positive lysosomes (Supplementary Figure 4). Next, we crossed *phmt*-*1::hmt*-*1::gfp* and *phmt*-1::*ABCB6::gfp* worms with *pges*-*1::mCherry::RAB*-*5, pges*-*1::mCherry::RAB*-*7, pges*-*1::mCherry::RAB*-*10* strains [25], which express the fluorescent mCherry protein in different endocytic compartments. Analysis of the transgenic strains showed that CeHMT-1 and ABCB6 partially colocalize with markers of the early, late and basolateral recycling endosomes (mCherry::RAB-5, mCherry::RAB-7 and mCherry::RAB-10, respectively) (Fig. 3b–d).

**Fig. 4:**
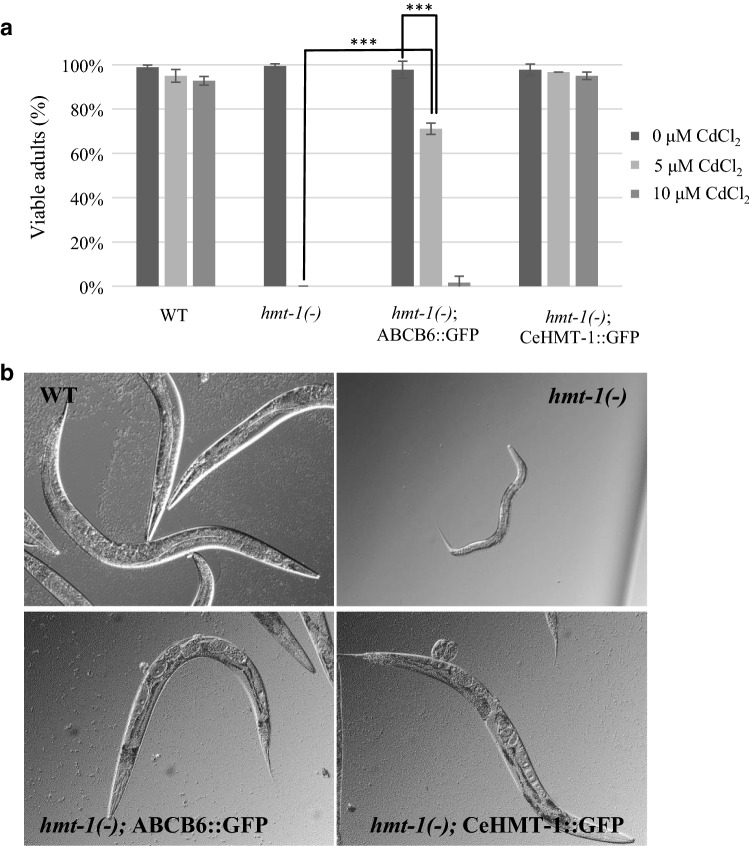
ABCB6 rescues the Cd-sensitive phenotype of HMT-1-deficient nematodes. **a** Wild-type (WT), *hmt*-*1*-deleted [*hmt*-*1(*−*)*], *hmt*-*1*-deleted expressing CeHMT-1::GFP [*hmt*-*1(*−*); hmt*-*1::gfp*] or ABCB6::GFP [*hmt*-*1(*−*); ABCB6::gfp*] adult hermaphrodites were placed individually onto NGM plates supplemented with the indicated concentrations of Cd, and were allowed to lay eggs for 2 h. Shown are the percentages of the progeny reaching adulthood 3 days after hatching (mean of 3 independent trials). ***: Student’s *T* test, *p* < 0,001; bars represent ± SD). **b** Representative pictures of animals grown on plates containing 10 µM CdCl_2_, 3 days after hatching at 20 °C. Heterologous expression of the human ABCB6 protein provided partial rescue, allowing the development of small sized adults, whereas *hmt*-*1*-deleted animals were arrested at the L2–L3 larval stages

Since the HMT-1 proteins in *S. pombe* and *C. elegans* have been shown to share an orthologous function [20], we investigated whether ABCB6 could also rescue the Cd-sensitive phenotype of an HMT-1-deficient *C. elegans* strain. Adult hermaphrodites were allowed to lay eggs onto NGM plates supplemented with the indicated concentrations of CdCl_2_, and the progeny reaching adulthood was counted 3 days after hatching at 20 °C (Fig. 4a). Whereas wild-type and HMT-1-deficient worms were indistinguishable in the absence of heavy metals, the latter were markedly more sensitive to Cd, showing developmental delay, larval arrest and death at early larval stages. As expected, expression of CeHMT-1 provided a full rescue. Remarkably, ABCB6 also restored tolerance to Cd exposure (Fig. 4a, b).

### Human ABCB6 confers Cd tolerance to SNB-19 glioblastoma cells

**Fig. 5:**
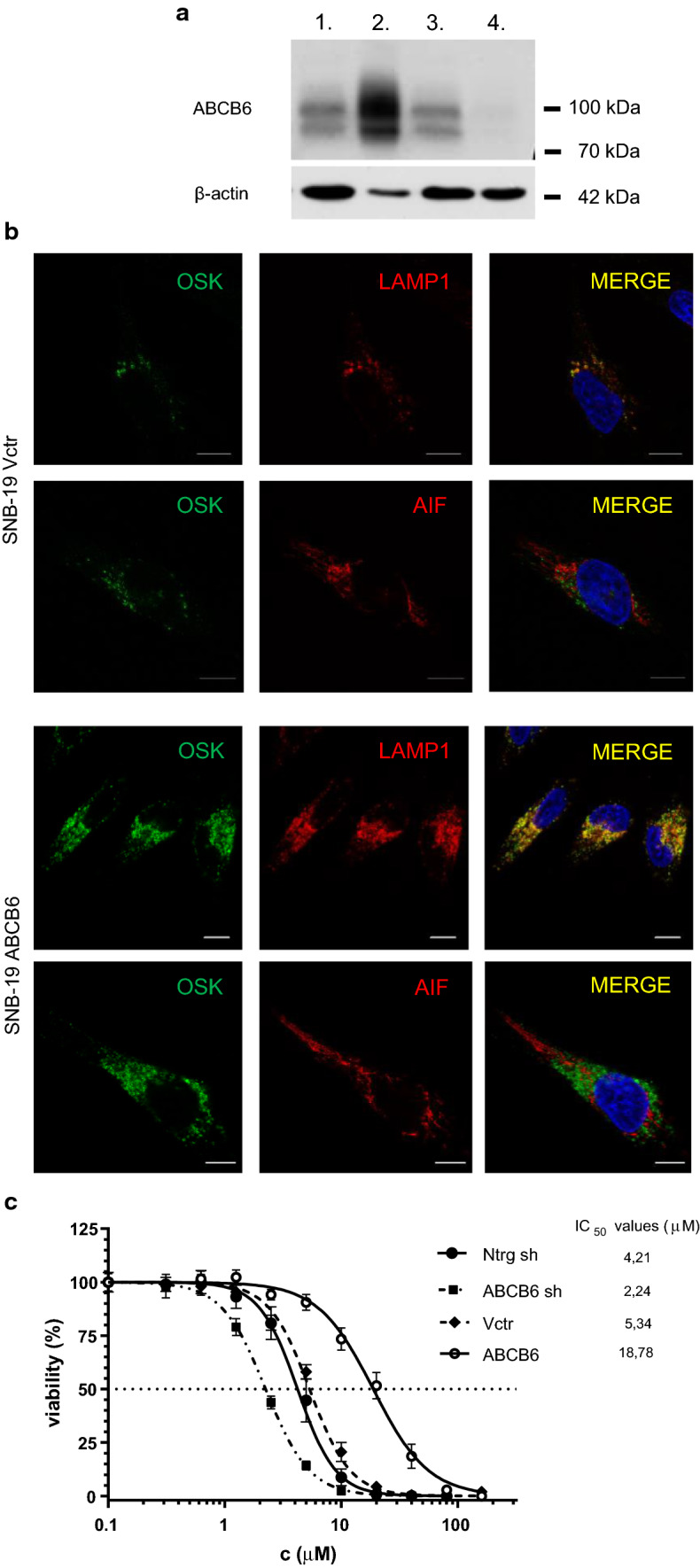
Differential expression of ABCB6 modulates Cd sensitivity of SNB-19 cells. **a** Overexpression and silencing of ABCB6 in SNB-19 cells. Differential expression of ABCB6 from total SNB-19 cell lysates was monitored by Western blotting, using the anti-ABCB6 antibody ABCB6-567 [11]. Whole cell lysates of SNB-19 cells (80 μg protein, lane 1), cells overexpressing ABCB6 (8 μg protein, lane 2), and cells transfected with a control (80 μg protein, lane 3) or an anti-ABCB6 shRNA vector (80 μg protein, lane 4). β-Actin is shown for loading control (see Supplementary Figure 5). **b** ABCB6 is expressed in the endolysosomal compartment of SNB-19 cells. Subcellular localization of endogenous and overexpressed ABCB6 was revealed by immunofluorescence labeling and laser scanning confocal microscopy. Endogenous (upper panels) and overexpressed (lower panels) ABCB6 was visualized using the OSK43 ABCB6 antibody (green); nuclei were labeled with Hoechst 33342 (blue); organelles were labeled with specific markers (red): mitochondria (AIF), lysosomes (LAMP1). Scale bar: 10 µm. **c** ABCB6 confers cadmium resistance in SNB-19 glioblastoma cells. SNB-19 cells were engineered to silence ABCB6 expression by ABCB6 shRNA (ABCB6 sh) or overexpress ABCB6 (ABCB6). As compared to cells expressing a non-target control shRNA (Ntrg sh) or a control (empty) vector (Vctr), overexpression of ABCB6 confers cadmium resistance, whereas attenuation of ABCB6 levels increases cadmium sensitivity. IC_50_ values represent means of triplicate measurements (see inset)

The functional relevance of ABCB6 in Cd sensitivity was further evaluated in SNB-19 glioblastoma cells. ABCB6 was overexpressed or silenced by lentiviral transduction (Fig. 5a, Supplementary Figure 5). Immunocytochemical analysis of SNB-19 cells by confocal microscopy confirmed the localization of the endogenous ABCB6 protein in the lysosomal compartment (labeled by LAMP1), and its absence in mitochondria (labeled by AIF) (Fig. 5b, upper panels). Overexpression of ABCB6 also resulted in endolysosomal expression that was clearly distinct from the mitochondrial pattern (Fig. 5b, lower panels). Attenuation of ABCB6 expression sensitized SNB-19 cells to Cd, as compared to cells stably transfected with the scrambled shRNA construct. In line with these results, ABCB6 overexpression conferred resistance to Cd, showing that ABCB6 effectively modulates the cadmium tolerance of SNB-19 cells (Fig. 5c).

## Discussion

Cadmium is a nonessential divalent metal ion, posing significant health concerns. Chronic exposure to cadmium is associated with increased mortality and cancer risk [26]. By displacing essential biological metals, cadmium induces oxidative stress and eventual cell death. Organisms have evolved several mechanisms to detoxify and eliminate cadmium from the cells [27]. In *Saccharomyces cerevisiae*, sequestration of cadmium–glutathione complexes is mediated by ScYCF1, which belongs to the ABCC subfamily [28]. In other species, vacuolar sequestration of cadmium complexes is mediated by HMT-1 proteins, which belong to the ABCB subfamily. Phylogenetic analysis shows that HMT-1s from *S. pombe*, *Arabidopsis thaliana*, *C. elegans* and *D. melanogaster* cluster with ABCB6, together with mitochondrial ATM proteins that are involved in iron-sulfur enzyme biogenesis. ABCB6 was initially named MTABC3, because it was considered to be the functional orthologue of Atm1P [4], leading to the erroneous classification of ABCB6 as a mitochondrial protein. Later studies have convincingly demonstrated that the functional orthologue of Atm1P is in fact ABCB7, which is a canonical mitochondrial ABC transporter localized to the inner mitochondrial membrane [29, 30].

The high degree of sequence and topological similarity between the HMT-1 proteins and ABCB6 suggests an evolutionary conserved function, implying ABCB6 in heavy metal resistance [19]. Circumstantial evidence including increased copy numbers, or increased expression in resistant cells [31–33], as well as correlation of gene expression to chemotherapy outcome has implicated ABCB6 in resistance to chemotherapeutic agents [34–36]. Overexpression of rat Abcb6 in LoVo cells conferred tolerance toward copper, suggesting an involvement of rAbcb6 in transition metal homeostasis [16]. There is a direct correlation between arsenic resistance and ABCB6 expression in various human and mouse cell lines, which was interpreted to be based on an ABCB6-mediated increase of cytosolic heme levels, resulting in the reduction of arsenite-induced oxidative stress [37–39]. However, models relying on the mitochondrial function of ABCB6 are difficult to reconcile with the endolysosomal expression pattern shown here and reported by several groups [12–17]. Cell fractionation experiments, images obtained of fixed cells with confocal and electron microscopy, and live cell imaging have repeatedly demonstrated that the endogenous ABCB6 protein is expressed in the endolysosomal system, and not in mitochondria.

*Schizosaccharomyces pombe* and *C. elegans* have served as important models for elucidating conserved pathways and processes relevant to human biology and disease. In particular, rescue of mutant phenotypes have established the function of several orthologous human proteins. In the past several years, well-developed genetic, genomic, biochemical and cell biological tools have provided fresh insights into vacuolar protein sorting, organelle homeostasis, autophagy, and stress-related functions of the yeast vacuole, and these insights have often found parallels in mammalian lysosomes [23, 40]. In this paper, we show that ABCB6 localizes to the same intracellular compartment as HMT-1, performing an overlapping function linked to the intracellular sequestration of metal complexes in both model organisms. Vacuolar localization in yeast was revealed by the expression of differently tagged ABCB6 and SpHMT-1 (Fig. 1b). In *C. elegans*, ABCB6 was expressed under the control of the endogenous CeHMT-1 promoter, offering an opportunity to study ABCB6 localization in an intact organism without the burden of artifacts associated with overexpression. In complete agreement with a recent report [41], we find that CeHMT-1 is localized to the endosomal compartment in the intestinal cells of the nematode (Fig. 3). Importantly, ABCB6 was found in the same intracellular compartment (Fig. 3). Our results confirm recent studies establishing the relevance of the N-terminal domain in the localization of ABCB6 and CeHMT-1 [13, 41]. Determining the subcellular localization of a protein is a key step toward understanding the cellular function of a protein. Although we find that the endogenous ABCB6 protein is confined to the endolysosomal compartment of SNB-19 glioblastoma cells, it may be argued that the precise intracellular localization can only be established with the discovery of a matching physiological function. In addition to evidence based on imaging of ABCB6 in native organisms, in this paper we provide functional proof supporting the role of ABCB6 in the vacuolar/endosomal sequestration of cadmium. First, we show that ABCB6 rescues the Cd-sensitive phenotype of HMT-1-deficient *S. pombe* and *C. elegans* strains. Second, we show that ABCB6 function is required for the sequestration of cadmium into HMT-1-deficient yeast vacuoles. Third, we provide evidence that ABCB6 modulates the cadmium sensitivity of human glioblastoma cells. Taken together, these results clearly establish ABCB6 as the human orthologue of HMT-1 proteins.

SpHMT-1 and CeHMT-1 confer cadmium resistance by sequestrating Cd–phytochelatin complexes. Phytochelatins (PCs) are (γ-Glu-Cys)n Gly polymers that are restricted to plants and fungi, with the notable exception of *C. elegans*. In animal cells and *S. cerevisiae*, cytoplasmic cadmium is complexed with glutathione (GSH), which is a common chelator involved in cellular response, transport and excretion of metal cations. Importantly, detoxification by CeHMT-1 does not depend on PC synthesis [21], and SpHMT-1 was shown to confer cadmium tolerance in the absence of phytochelatins, but depending on the presence of GSH and ATP, demonstrating that a common, highly conserved mechanism has been selected during evolution [20, 42]. Given the conservation of HMT-1 proteins, we suggest that the ABCB6-mediated increase of vacuolar cadmium levels shown in Fig. 2 can be explained by the direct transport of (Cd–GS_2_) complexes. Remarkably, overexpression of ABCB6 conferred resistance to cadmium in human SNB-19 cells, suggesting that the HMT-1 detoxification pathway is preserved from yeast to human. The contribution of GSH to cadmium detoxification was further suggested by experiments in which SNB-19 cell overexpressing ABCB6 depleted of GSH showed an increased cadmium sensitivity (not shown here). However, no resistance against cadmium has been observed in HeLa cells overexpressing ABCB6 (not shown here). In that respect, ABCB6 is similar to ABCC1 (MRP1), whose role in cadmium detoxification appears to be cell specific [42], even though it can functionally complement ScYCF1 in yeast [43]. The reason why the orthologous function of ABCB6 (and ABCC1) does not uniformly manifest in all mammalian cell models is not clear. In mammals, cadmium detoxification relies primarily on metallothioneins, which bind Cd and also scavenge free radicals generated in oxidative stress [44]. Also, we cannot rule out that the role of ABCB6 in cadmium detoxification of mammalian cells is indirect. Whereas mitochondrial uptake of porphyrins seems improbable, ABCB6 may mediate the sequestration of toxic by-products of Cd–heme interactions into the endolysosomal system [41]. In all examined organisms, overexpression of SpHMT-1 conferred tolerance to cadmium, but not to Sb(III), Ag(I), As(III), As(V), Cu(II), or Hg(II) [42]; whereas substrates of CeHMT-1 also include As(III) and Cu(II) [21], indicating how subtle changes in the primary sequence of transporters can fine-tune substrate specificity through evolution [45]. In fission yeast, ABCB6 conferred resistance to Cd(II), As(III), but not to As(V) or Cu(II) (Supplementary Figure 2). Preliminary experiments using purified ABCB6 protein have failed to demonstrate stimulation of the ABCB6 ATPase activity by cadmium–GSH complexes (not shown here). Future work, using reconstituted, transport-competent ABCB6 will be needed to verify the exact nature and extent of ABCB6 substrates.

It also remains to be determined how the evolutionary conserved role in detoxification is manifested in pathological conditions associated with impaired ABCB6 function. Interestingly, lack of ABCB6 in mice does not result in an overt phenotype [12, 46], and ABCB6 deficiency in humans, as observed in Lan-negative individuals, is also without any clinical consequences [5]. On the other hand, disruption of the *ABCB6* gene in mice exacerbates porphyria phenotypes in the Fech(m1Pas) mouse model [47], and ABCB6 is a genetic modifier of porphyria [47]. Mutations in the *ABCB6* gene were implied in several hereditary diseases ranging from pseudohyperkalemia, coloboma [48], or dyschromatosis universalis hereditaria (DUH) [7, 49, 50]. The pathogenic role of ABCB6 in these conditions is not understood, as there is no obvious overlap between these phenotypes. Pseudohyperkalemia is a dominant red cell trait characterized by increased serum [K^+^] in whole blood stored at, or below room temperature (RT), without additional hematological abnormalities [51]. Coloboma is a developmental disorder affecting the eyes, whereas DUH is characterized by asymptomatic hyper- and hypopigmented macules distributed over the body. Based on the results presented in this study, it is tempting to speculate that a common theme in these phenotypes may be disturbed endolysosomal metal homeostasis due to the impaired sequestration of glutathione adducts. The relevance of the endolysosomal compartment in the metabolism/homeostasis of metals is well-known [52]. Thus, the coloboma phenotype may be related to the pathophysiological consequences associated with cadmium exposure, which was shown to alter visually guided behavior in zebrafish as a result of toxicity occurring at the cellular level [53]. Similarly, the ultrastructural abnormalities observed in MNT-1 cells expressing DUH mutant ABCB6 variants may be explained by the impaired intraluminal homeostasis of the maturing early melanosome [12].

The identification of ABCB6 as an HMT-1 orthologue links ABCB6 to heavy metal-related diseases, such as neurodegenerative conditions, dysfunction of the digestive tract and cancer [19]. The pathophysiological relevance of ABCB6 in these conditions remains to be confirmed by studies using relevant disease models. In parallel, heterologous expression of ABCB6 in *hmt*-*1*-deficient *S. pombe* cells may be used as a tool for better understanding the structure and function of ABCB6.

## Materials and methods

### Cell culturing

#### *S. pombe* culture conditions and strains

The *S. pombe* wild-type strain BG_00008 (ade6-M216, ura4-D18, leu1-32) and the *hmt*-*1*-deleted mutant strain BG_H4691 (ade6-M216, ura4-D18, leu1-32) was a generous gift from R. Lill (Philipps-Universität Marburg). Edinburgh Minimal Medium (EMM Broth, EMM agar and EMM without dextrose) were obtained from Formedium (Hunstanton, UK).

#### *C. elegans* culture conditions and strains

*Caenorhabditis elegans* strains were maintained at 20 °C on solid Nematode Growth Medium (NGM) using the *E. coli* OP50 strain as a food source [54]. The following strains were used: N2 *C. elegans* wild-type, var. Bristol; DP38 *unc*-*119(ed3)III*; VC287 *hmt*-*1(gk161)III*; VF31 *gfIs1[phmt*-*1::hmt*-*1::gfp, unc*-*119(*+*)*; VF12 *hmt*-*1(gk161)III; gfIs1[phmt*-*1::hmt*-*1::GFP, unc*-*119(*+*)]. XW1957: qxIs110 (pges*-*1::mCHERRY::RAB*-*5); XW1962: qxIs111 (pges*-*1::mCHERRY::RAB*-*7); XW9119: qxIs213 (pges*-*1::mCHERRY::RAB*-*10)* strains were a kind gift from Dr. Xiaochen Wang (Institute of Biophysics, Chinese Academy of Sciences).

#### Cell lines

The SNB-19 glioblastoma cell line was obtained from DSMZ (Germany), HeLa cells were from ATCC. Cells were grown in high glucose DMEM (Gibco 521000-47) supplemented with 10% FBS, 2 mmol/L glutamine, and 100 units/mL penicillin and streptomycin (Life Technologies) at 37 °C in 5% CO_2_. Cells were periodically tested for mycoplasma contamination with the MycoAlert mycoplasma detection Kit (Lonza, Basel, Switzerland).

### Molecular cloning of *ABCB6* and *hmt*-*1* constructs

#### *Schizosaccharomyces pombe*

Plasmid constructs were amplified in *E. coli* strain Top10 (Invitrogen, Carlsbad, CA, USA) grown at 37 °C in liquid Luria–Bertani (LB) medium supplemented with appropriate antibiotics. Hemagglutinin-tagged *S. pombe hmt*-*1* (Z14055) cDNA was synthesized by GenScript (Piscataway, NJ, USA). Site-specific mutation was engineered using the QuikChange site-directed mutagenesis kit (Stratagene, San Diego, CA, USA); the mutation was confirmed by sequencing. The cDNAs encoding *hmt*-*1* and *ABCB6* variants were subcloned into the pREP1 fission yeast expression vector; pEGFP-N1 (BD Biosciences, Franklin Lakes, NJ, USA) was used for the N-terminal EGFP-tagging of the transporters.

#### *Caenorhabditis elegans*

To generate the p*hmt*-*1::ABCB6::gfp* and p*hmt*-*1::ABCB6::mCherry* reporter, codon-optimized *ABCB6* cDNA was synthesized by GenScritpt (Piscataway, NJ, USA). After restriction digestion with *SphI* and *XmaI*, *ABCB6* was subcloned in frame with the *gfp* sequence of the pPD95.75 vector. A 5′ regulatory region of *hmt*-*1* (2.8 kb immediately upstream of the start of the *hmt*-*1* coding sequence) was PCR-amplified using primer pairs designed to introduce *SphI* and *HpaI* restriction enzyme recognition sites at the 5′ and 3′ ends, respectively. The PCR-amplified *hmt*-*1* promoter was subcloned into the pPD95.75-ABCB6-EGFP vector. In the case of p*hmt*-*1*::*ABCB6::mCherry*, the GFP reporter sequence of pPD95.75-ABCB6-EGFP was changed to the sequence encoding mCherry.

### Generation of cell lines

#### Yeast transgenic strains

Yeast cells were grown at 30 °C in Edinburgh minimal medium (EMM). At an *A*_600nm_ (OD600) of 1, cells were grown in EMM containing minimal glucose (5 g/L). Cells were transformed using the standard lithium acetate procedure [55]. *S. pombe* transformants were selected for leucine prototrophy in EMM.

#### *C. elegans* transgenic strains

Transgenic *C. elegans* strains were generated by biolistic transformation using the Biolistic PDS-1000/He particle delivery system (BioRad, Hercules, CA, USA) according to standard methods described by Rieckher et al. [56]. Briefly, 10-10 μg linearized *phmt*-*1*::*ABCB6::gfp* and *phmt*-*1::ABCB6::mCherry* reporter plasmids, together with pRH21[*unc*-*119(*+*)*] co-transformation marker plasmid DNA were bombarded onto *unc*-*119(ed3)* mutant adult hermaphrodites. Non-Unc transgenic animals exhibiting GFP or mCherry-mediated fluorescence were selected, and the stable integrated strains TTV634: *eluIs310 [phmt*-*1::ABCB6::gfp *+* unc*-*119(*+*)]; unc*-*119(ed3)* and TTV677: *eluEx383[phmt*-*1::ABCB6::mCherry *+* unc*-*119(*+*)]; unc*-*119(ed3)* carrying the array extrachromosomally were selected for further analysis. TTV 634 was crossed with *hmt*-*1(gk161)* to generate TTV 635: *hmt*-*1(gk161); eluIs310 [phmt*-*1::ABCB6::gfp *+* unc*-*119(*+*)].*

#### Human cell lines with enhanced or silenced ABCB6 expression

*ABCB6* knock-down and overexpression were achieved using a self-inactivating lentiviral system, as described previously in [9]. To induce the expression of the shRNA constructs, IPTG (1 mM) was added to the cells for 6 days before additional treatments.

### Immunoblotting

Overnight cultures were grown to *A*_600nm_ of 1.5–2. Immunoblotting of human cell lines was performed according to standard protocol. The following primary monoclonal antibodies were used in Western blotting experiments: β-actin (A1978, Sigma-Aldrich, Saint Louis, MO, USA); anti-EGFP (ab184601 Abcam, Cambridge, UK), ABCB6-567 [11], anti-HA antibody, (H6908 Sigma-Aldrich). The HRP-dependent luminescence was detected using the enhanced chemiluminescence technique (ECL, Amersham).

### Cytotoxicity assays

#### *Schizosaccharomyces pombe*

Transformed cells were grown in EMM complemented with appropriate supplements. To characterize the chemosensitivity of yeast strains in liquid medium, 100-μL overnight cultures (*A*_600nm_ of 0.8) were diluted into 2-mL EMM containing different concentrations of metal compounds (Cd(II) As(III) As(V), Sb(III), Sb(V), Hg(II), Cu(II), or Zn(II)). In case of Sb(III) and Cu(II), we could not detect toxic concentrations in EMM medium. Cells were then grown at 30 °C. The extent of growth after 72 h was determined by measuring absorbance at 600 nm (*A*_600nm_). Viability curves were fitted with Graph Pad Prism 5 software using the sigmoidal dose–response model. To characterize chemosensitivity on agar plates, overnight cultures were diluted in EMM (*A*_600nm_ of 0.7). Colonies were spotted onto plates containing different concentrations of metal compounds and incubated for 6–7 days at 30 °C.

#### *Caenorhabditis elegans*

Heavy metal tolerance of *C. elegans* strains was assayed as described in [19]. Briefly, 8–10 adult worms were allowed to lay eggs for 2 h at 20 °C on NGM plates supplemented with the indicated concentrations of CdCl_2_. CdCl_2_ tolerance was quantified by determining the ratio of adult worms and larvae after 3 days at 20 °C. At least 60 animals, derived from 3 parallel plates containing at least 20 animals/category, were counted by light microscopy in 3 independent trials.

#### Human cell lines

In cytotoxicity experiments, cells were seeded in 100-μL DMEM medium at a density of 4000 cells/well in 96-well plates, and serially diluted drugs were added on the following day in 100-μL medium to give the indicated final concentration. Cells were then incubated for 72 h at 37 °C in 5% CO_2_. Cytotoxicity assays were performed in triplicate. Cell survival was assessed by the PrestoBlue assay (Life Technologies), according to the manufacturer’s instructions. Viability curves were fitted with Graph Pad Prism 5 software using the sigmoidal dose–response model.

### Determination of the vacuolar cadmium content

#### Vacuole isolation

*Schizosaccharomyces pombe* cultures were treated overnight with 20 μM CdCl_2_. Vacuoles were isolated as described in [20], with some modifications. Briefly, 5-mL stationary phase cultures diluted in 25 mL of EMM were grown for 4–6 h at 30 °C. Next, 25-mL cultures were diluted in 200-mL EMM medium containing 20-μM CdCl_2_, and the cultures were grown for 18 h at 30 °C to an *A*_600nm_ of 1.5. Cells were pelleted by centrifugation at 3000×*g* for 5 min, and were washed in 50-mL distilled water. After resuspension in 50-mL buffer (20-mM β-mercaptoethanol, 100-mM Tris–HCl, pH 9.4), cells were incubated for 20 min at 30 °C with gentle shaking. Spheroplasts were created by pelleting and resuspending the cells in 20 mL of digestion medium (1.2-M sorbitol, 10-mM β-mercaptoethanol, 20-mM potassium phosphate, 50 mg of Zymolyase 20T (ICN) and 100 mg of lysing enzymes from *Trichoderma harzianum* (Sigma-Aldrich), pH 7.5). The suspension was incubated for 2 h at 30 °C with gentle shaking, followed by centrifugation at 3000×*g* for 5 min. The spheroplasts were washed in 20-mL ice-cold homogenization medium (1.6-M sorbitol, 10-mM MES–Tris, 0.5-mM MgCl_2_, 5-mM β-mercaptoethanol, 1-mM phenylmethylsulfonyl fluoride, and 1 µg/mL each of leupeptin, aprotinin, and pepstatin (protease inhibitor cocktail, Sigma P8340 in 500× dilution), pH 6.9). Pelleted spheroplasts were lysed in the same medium by homogenization in a 5-mL glass Dounce homogenizer. The crude lysate was cleared of cell debris and unbroken cells by centrifugation at 3000×*g* for 8 min. The supernatants were collected and the pellet was resuspended in 3.5-mL HM, homogenized again (30×), and centrifuged at 3000×*g* for 8 min. Supernatants were centrifuged at 13,000×*g* at 4 °C for 35 min. The pellet, containing the partially purified vacuolar fraction was suspended in 1.5 mL HM, layered onto 1-mL Sucrose step gradient (40%/50% (v/v)), and pelleted at 40,000 rpm using a Beckman Coulter 70.1 Ti rotor at 4 °C for 1 h. Purified vacuoles were suspended in 3-mL suspension medium (1.6-M sorbitol, 100-mM KCl, 10-mM MES-Tris, 5-mM MgCl_2_, and protease inhibitors pH 6.9), and were centrifuged at 4 °C at 13,000×*g* for 12 min in an Eppendorf microcentrifuge. The final vacuolar pellet was stored at − 80 °C.

#### Assessment of integrity of vacuole preparations

The integrity of the vacuoles was assessed by measuring fluorescence as described in [57]. Acridine orange (AO, Sigma-Aldrich) fluorescence (Supplementary Figure 3) was measured using an Attune Acoustic Focusing cytometer (Applied Biosystems, Life Technologies, Carlsbad, CA, US).

#### Determination of vacuolar Cd contents

Vacuolar Cd content was determined by graphite furnace atomic absorption spectrometry (GFAAS). All GFAAS measurements were performed on an Analytik Jena Model ContrAA-700 tandem high-resolution AAS spectrometer (Analytik Jena AG, Jena, Germany), equipped with an MPE-60 autosampler. Each final vacuolar pellet after the isolation was digested in 200-µL cc. (65% v/v) HNO_3_ for 24 h at RT. After appropriate dilution, an aliquot of 20 µL of each sample was directly dispensed by the autosampler into the pyrolytic graphite coated graphite tube (fitted with a pyrolytically-coated graphite platform) to determine the concentration of Cd in the isolated vacuoles. The Cd 228.8018 nm spectral line was selected for the determinations, with a 3-pixel evaluation of the CCD camera, which corresponds to a resolution of 3.78 pm at this wavelength. The GF heating program consisted of smooth drying (at 100 °C for 20 s, 110 °C for 5 s, 130 °C for 10 s), pyrolysis (350 °C for 15 s, 450 °C for 10 s), atomization/measurement (at 1200 °C for 3 s), and clean-out (2450 °C for 4 s) steps. In these steps, the maximum flow (2 dm^3^/min) of the GF sheath gas (5.0 Ar, supplier: Messer, Hungary) was applied, except the atomization step, being set to stopped gas flow. Integrated, 3D-absorbance signals were recorded with integration time of 3 s, using iterative spectral background correction. Each measurement data corresponds to an average of three replicate determinations. For quantitative determinations, external standardization was applied by means of setting up five-point calibration curves (range: 0.5–50 ng/mL Cd; solutions preserved in 2.6% (v/v) HNO_3_). Recovery was checked by spiking selected samples with 5 µL of a Cd standard solution at a concentration of 5 ng/mL and 50 ng/mL. The precision of the determinations, expressed as relative standard deviation (RSD), was typically below 2.1%, but never worse than 5.3%. All Cd concentration data were normalized to the protein content of the samples.

### Confocal microscopy

#### Localization of ABCB6 in *S. pombe*

For the evaluation of intracellular localization of the transporters, *hmt*-*1*-deleted *S. pombe* was transformed with pREP1-HMT-1-GFP or ABCB6-GFP. Cells were grown to mid-log phase (*A*_600nm_ of 0.5–0.8) and stained with FM 4–64 as described in [58] with the following modifications. FM 4–64 (T3166 ThermoFischer Scientific Waltham, MA, USA) was dissolved in dimethyl-sulphoxide at a concentration of 1.64 mM. Cells were harvested and incubated with 1-μL FM 4–64 in 50-μL EMM medium at 30 °C for 20 min. An aliquot of 1-mL EMM was added and cells were centrifuged at 5000×*g* for 5 min at RT. The cell pellet was resuspended in 5-mL EMM, and the suspension was shaken at 30 °C for 90 min. The total volume was transferred to a centrifuge tube and spun for 5 min at 5000×*g* at RT. The cell pellet was resuspended in 1-mL sterile water, and centrifuged at 5000×*g* for 5 min at RT. Cells were resuspended in 25-μL EMM. An aliquot of 7 μL was spotted on ConA/polyK-coated (1:1 mixture of 2 mg/mL concanavalin A and 0.1% poly-l-lysine) glass slides covered with an 18 × 18 mm^2^ cover slip. Confocal images were obtained using a LSM 710 confocal laser scanning microscope (Carl Zeiss AG, Oberkochen, Germany) equipped with a Plan-Apochromat 63 ×/1.4 Oil DIC M27 objective. Noise reduction and deconvolution of the images were performed with Huygens Essential (Scientific Volume Imaging B.V.).

#### Localization of ABCB6 in *C. elegans*

Transgenic strains were grown in normal growth conditions at 20 °C. To test the subcellular co-localization of ABCB6 and CeHMT-1, the *phmt*-*1::ABCB6::mCherry* (TTV677) strain was crossed with *phmt*-*1::HMT*-*1::GFP* (VF31) males and the F1 progeny co-expressing both transgenes was examined with a confocal microscope (Zeiss LSM 710, Plan-Apochromat 63 ×/1.4 NA Oil DIC M27objective). Lysosomal staining was performed as described in [24]. To determine the subcellular localization of CeHMT-1::GFP and ABCB6::GFP, *phmt*-*1::hmt*-*1::gfp* (VF31) and *phmt*-*1::ABCB6::gfp* (TTV634) were crossed with strains expressing different endosomal markers [25], resulting in *TTV701 unc*-*119(ed3)III; eluIs310[phmt*-*1::ABCB6::gfp *+* unc*-*119(*+*)]; qxIs110(Pges*-*1mCHERRY::RAB*-*5), TTV702 unc*-*119(ed3)III; eluIs310[phmt*-*1::ABCB6::gfp *+* unc*-*119(*+*)];* qxIs111*(Pges*-*1mCHERRY::RAB*-*7), TTV703 unc*-*119(ed3)III; eluIs310[phmt*-*1::ABCB6::gfp *+* unc*-*119(*+*)]; qxIs213(Pges*-*1mCHERRY::RAB*-*10), TTV705 unc*-*119(ed3)III; gfIs1[phmt*-*1::hmt*-*1::gfp, unc*-*119(*+*)]; qxIs110(Pges*-*1mCHERRY::RAB*-*5), TTV706 unc*-*119(ed3)III; gfIs1[phmt*-*1::hmt*-*1::gfp, unc*-*119(*+*)];* qxIs111*(Pges*-*1mCHERRY::RAB*-*7), TTV707 unc*-*119(ed3)III; gfIs1[phmt*-*1::hmt*-*1::gfp, unc*-*119(*+*)]; qxIs213(Pges*-*1mCHERRY::RAB*-*10)*.

#### Localization of ABCB6 in human cells

Monoclonal antibodies, dyes and their sources were as follows: Rabbit monoclonal Anti-AIF [D39D2] antibody (#5318) to apoptosis inducing factor, rabbit monoclonal Anti-EEA1 [C45B10] antibody (#3288) to early endosome antigen 1, rabbit monoclonal Anti-LAMP1 [D2D11] antibody (#9091) to lysosome-associated membrane protein 1, secondary goat anti-mouse IgG (H + L) F(ab′)_2_ fragment conjugated to Alexa Fluor 647 (#4410) and secondary goat anti-rabbit IgG (H + L) F(ab′)_2_ fragment conjugated to Alexa Fluor 647 (#4414) were from Cell Signaling Technology. Secondary goat polyclonal antibody to human IgG conjugated to DyLight 488 (ab96907) was purchased from Abcam. Hoechst 33342 (R37605) nuclear counterstain was from Thermo Fisher Scientific. The OSK43 antibody was a kind gift from Dr. Yoshihiko Tani (Japanese Red Cross Osaka Blood Center, Osaka, Japan). SNB-19 cells expressing ABCB6 variants were plated in an Eppendorf 8-well imaging coverglass (#0030742036). Hoechst 33342 was applied to the cells for 20 min prior to fixation; subsequently, cells were rinsed in PBS and fixed for 30 min in 4% Paraformaldehyde/PBS at RT. Fixed cells were quenched for 10 min in PBS/100 mM glycine (quenching buffer), washed with PBS and blocked and permeabilized in PBS containing 0.2 mg/mL BSA/0.1% Triton X-100/10% Normal Goat Serum (blocking buffer). Primary antibody was diluted in PBS containing 0.2 mg/mL BSA/0.1% Triton X-100/3% normal goat serum (incubation buffer, IB). Cells were incubated with the primary antibody overnight at 4 °C in a humified chamber, washed five times in IB, and incubated with the corresponding secondary anti-human, anti-rabbit and anti-mouse antibodies conjugated to Alexa Fluor 488 or Alexa Fluor 647 diluted in IB for 90 min at RT. Samples were washed five times with PBS and subsequently imaged. Confocal images were obtained using a LSM 700 confocal laser scanning microscope (Carl Zeiss, Inc.) equipped with a Plan-Apochromat 63×/1.4 NA Oil DIC M27 objective. Images were acquired in three channels (blue (Hoechst33342), green (Alexa Fluor 488), red (Alexa Fluor 647)), blue emitting Hoechst 33342 was excited using the 405 nm laser line, green emitting Alexa Fluor 488 was excited using the 488 nm laser line and infrared emitting Alexa Fluor 647 was excited using the 633 nm laser line. Noise reduction and deconvolution of the images were performed with Huygens Essential (Scientific Volume Imaging B.V.).

### Electronic supplementary material

Below is the link to the electronic supplementary material.

Supplementary material 1 (DOCX 391 kb)
