# Supplementary material for: The human ABCB6 protein is the functional homologue of HMT-1 proteins mediating cadmium detoxification
Source: Cell Mol Life Sci. 2019 May 3;76(20):4131–44. doi: 10.1007/s00018-019-03105-5 (PMC6785578; doi:10.1007/s00018-019-03105-5)

**The human ABCB6 protein is the functional orthologue of HMT-1 proteins mediating cadmium detoxification**

Zsófia Rakvács^1^, Nóra Kucsma^1^, Melinda Gera^1^, Barbara Igriczi^1^, Katalin Kiss^1^, János Barna^2^, Dániel Kovács^2^, Tibor Vellai^2^, László Bencs^4^, Johannes M. Reisecker^5^, Norbert Szoboszlai^3^ and Gergely Szakács^1,5,*^

**^1^**Institute of Enzymology, Research Centre for Natural Sciences, Hungarian Academy of Sciences, Budapest, Hungary;

**^2^**Institute of Biology, Department of Genetics, Eötvös Loránd University, Budapest, Hungary;

**^4^**Institute for Solid State Physics and Optics, Wigner Research Centre for Physics, Hungarian Academy of Sciences, Budapest, Hungary

**^5^**Institute of Cancer Research, Department of Medicine I, Comprehensive Cancer Center, Medical University of Vienna, Vienna, Austria

*Corresponding author: gergely.szakacs@meduniwien.ac.at

**Supporting Information Legends**

**Supplementary Table 1.** Clustal2.1 Percent Identity Matrix.

Sequence similarity was quantified using the NCBI Blastp algorithm (<https://blast.ncbi.nlm.nih.gov/BlastAlign.cgi>).

|  | SpHMT-1 | CeHMT-1 | DmHMT-1 | ABCB6 |
| --- | --- | --- | --- | --- |
| SpHMT-1 | 100.00 | 35.68 | 40.42 | 39.20 |
| CeHMT-1 | 35.68 | 100.00 | 45.69 | 46.19 |
| DmHMT-1 | 40.42 | 45.69 | 100.00 | 50.43 |
| ABCB6 | 39.20 | 46.19 | 50.43 | 100.00 |

**Supplementary Figure 1. ABCB6 confers cadmium tolerance in *hmt-1*Δ mutant *S. pombe***

Wild-type *S. pombe* cells transformed with empty pREP1 vector (WT); *hmt-1*Δ mutant cells transformed with empty pREP1 vector (*hmt-1*Δ), pREP1-HMT-1-HA (*hmt-1*Δ/SpHMT-1-HA), pREP1-ABCB6 (*hmt-1*Δ/ABCB6) or pREP1-ABCB6-KM (*hmt-1*Δ/ABCB6-KM) overnight cultures were diluted (*A*_600nm_ of 0.7) then spotted onto solid EMM supplemented with adenine, uracil and the indicated concentrations of CdCl_2_. Colonies were visualized after incubating the plates for 3, 6 or 7 days at 30°C. At day 3 (left panel), the difference between the growth of WT and ABCB6-complemented cells on 50 μM CdCl_2_ appears to be bigger. However, by days 6 and 7, the ABCB6-mediated rescue of *hmt1*-deleted cells is evident (middle, right panel).

**Supplementary Figure 2. ABCB6 mediates resistance to As(III) in *hmt-1*Δ mutant *S. pombe***

Transformants were grown overnight to an *A*_600nm_ of 0.8-1. 100 µL aliquots were inoculated into 2 mL of the same medium containing the indicated concentrations of metal complexes. Absorbance was measured after growth at 30°C for 48 h. Values, expressed as viability (%), were normalized to untreated control (n=2)

**Supplementary Figure 3. Assessment of vacuolar integrity**

The integrity of the vacuoles was assessed by measuring fluorescence following incubation with 30 μM Acridin-Orange (AO, Sigma-Aldrich) for 10 min (red histograms). In control experiments (blue histograms), vacuoles were treated with 0,1% TritonX for 10 min after AO staining. Fluorescence was measured using an Attune Acoustic Focusing cytometer.

**Supplementary Figure 4. ABCB6 and CeHMT-1 are not expressed in the lysosomes of *C. elegans*.**

Lysosomal staining of strains expressing CeHMT-1::GFP or ABCB6::GFP was performed as described [24]. The panels show the DIC images (left) the GFP (green) and the Lysotracker (red) signals and the overlay of the two (right). Scale bar: 20 μm.

**Supplementary Figure 5. Densitometry of ABCB6 expression in human SNB-19 cell lines**

Quantification of relative ABCB6 contents based on the densitometry of Western blots developed by anti-ABCB6 (top panel) and anti-β-actin (lower panels) antibodies. The intensity of the bands was quantified using Image J, the values shown represent the ABCB6/β-actin ratio.

**Supplementary Figure 1.**


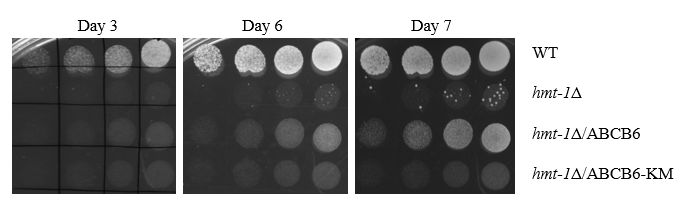


**Supplementary Figure 2.**


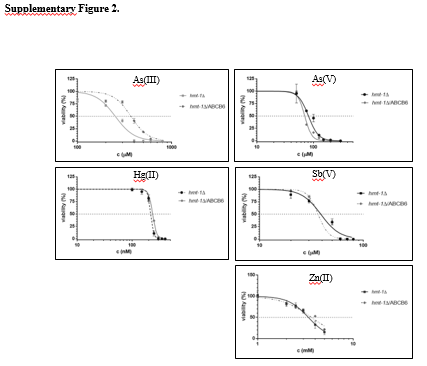


**Supplementary Figure 3.**


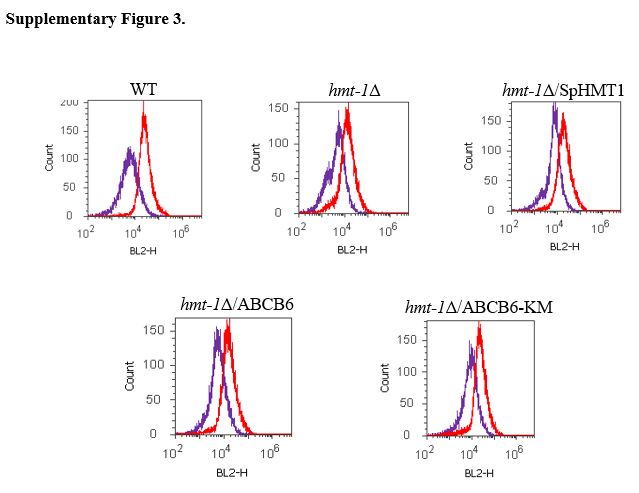


**Supplementary Figure 4.**


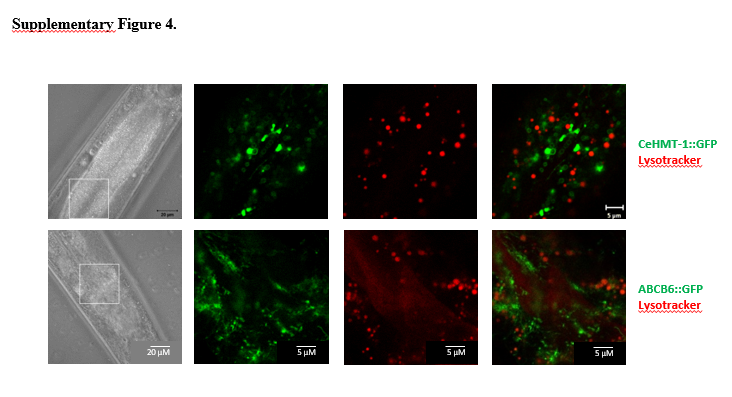


**Supplementary Figure 5.**


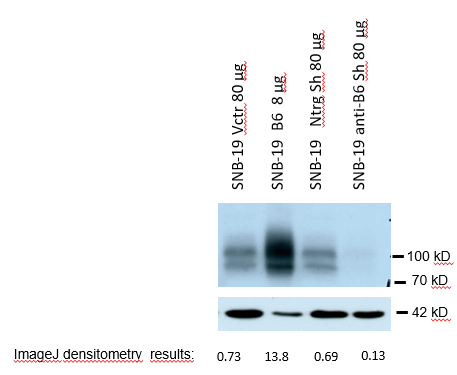

Supplement: Supplementary file 1 — Supplementary material 1 (DOCX 391 kb) [file 18_2019_3105_MOESM1_ESM.docx]
